# Characteristics and outcomes of patients with a history of cancer recruited to heart failure trials

**DOI:** 10.1002/ejhf.2818

**Published:** 2023-03-15

**Authors:** Stephen J.H. Dobbin, Li Shen, Mark C. Petrie, Milton Packer, Scott D. Solomon, John J.V. McMurray, Ninian N. Lang, Pardeep S. Jhund

**Affiliations:** ^1^ BHF Glasgow Cardiovascular Research Centre, Institute of Cardiovascular and Medical Sciences University of Glasgow Glasgow UK; ^2^ Department of Medicine Hangzhou Normal University Hangzhou China; ^3^ Department of Clinical Sciences University of Texas Southwestern Medical Center Dallas TX USA; ^4^ Division of Cardiovascular Medicine Brigham and Women's Hospital Boston MA USA

**Keywords:** Cancer, Cardiovascular, Clinical outcomes, Heart failure, HFpEF, HFrEF

## Abstract

**Aims:**

Heart failure (HF) therapy trials usually exclude cancer patients. We examined the association between cancer history and outcomes in trial participants with HF and reduced (HFrEF) or preserved ejection fraction (HFpEF).

**Methods and results:**

We combined PARADIGM‐HF and ATMOSPHERE, which enrolled HFrEF patients (*n* = 15 415) and we pooled HFpEF patients (ejection fraction ≥45%) enrolled in PARAGON‐HF and CHARM‐Preserved (*n* = 7363). The associations between cancer history, cardiovascular (CV) death, HF hospitalization, non‐CV and all‐cause death in these trials were examined. Incident cancer diagnoses during these trials were also measured. There were 658 (4.3%) and 624 (8.5%) patients with a cancer history in the HFrEF and HFpEF trials, respectively. HFrEF patients with a cancer history had a higher risk of HF hospitalization (adjusted hazard ratio [HR] 1.28; 95% confidence interval [CI] 1.07–1.52, *p* = 0.007) and non‐CV death (adjusted HR 1.57; 95% CI 1.16–2.12, *p* = 0.003) than those without. The risks of other outcomes were similar. There were no differences in the risk of any outcome in HFpEF patients with and without a cancer history. Adjusting for age and sex, the incidence of new cancer in the HFrEF and HFpEF trials was 1.09 (95% CI 0.83–1.36) and 1.07 (95% CI 0.81–1.32) per 100 person‐years, respectively.

**Conclusions:**

Although participants in HFrEF trials with a cancer history had higher risks of HF hospitalization and non‐CV death than those without, the risks of CV and all‐cause death were similar. Outcomes in HFpEF patients with and without a cancer history were similar. Incident cancer diagnoses were similar in HFrEF and HFpEF trials.

## Introduction

More than 50% of patients now survive longer than 10 years following a diagnosis of cancer.^1^ As these patients are living longer, they are at risk of developing age‐related conditions such as heart failure (HF). Furthermore, the risk of developing HF may be amplified by agents used to treat cancer.^2^ In patients with cancer, the subsequent lifetime risk of cardiovascular (CV) disease may be greater than the residual risk of the initial malignancy or its recurrence.^3^ Therefore, these patients are an increasingly relevant group for whom a clear understanding of the competing risks of CV and cancer outcomes is essential to inform treatment decisions.

In a large Danish administrative database, 17% of patients with HF also had a history of cancer. Malignancy was less frequent in age‐ and sex‐matched controls without HF.^4^ In a cohort of almost 10 000 American patients, 19.5% had a history of cancer.^5^ Despite this, patients with a history of recent or active cancer have either been excluded from or under‐represented in clinical trials in HF. This is because the competing risk of death from cancer has been viewed as being excessively large and not modifiable by therapies designed to improve CV outcomes. Although this argument has been used to justify the exclusion of patients with a history of cancer from trials, it is unclear whether this might now be inappropriate. This may be especially relevant to HF trials. Outcomes for patients with HF remain poor, if not poorer, than for many common cancers.^6^, ^7^

We examined the association between prior cancer and outcomes in trials enrolling patients with HF and reduced (HFrEF) or preserved ejection fraction (HFpEF). We also examined the reporting of new cancer diagnoses in these trials.

## Methods

### Study populations

Patients from two large, randomized trials in patients with HFrEF and HFpEF were included. The design and results of PARADIGM‐HF (Prospective comparison of ARNI with ACEI to Determine Impact on Global Mortality and morbidity in Heart Failure), ATMOSPHERE (Aliskiren Trial to Minimise Outcomes in Patients with Heart Failure), PARAGON‐HF (Prospective comparison of ARNI with ARB Global Outcomes in Heart Failure with Preserved Ejection Fraction) and CHARM‐Preserved (Effects of Candesartan in Patients with Chronic Heart Failure and Preserved Ejection Fraction) are published.^8^, ^9^, ^10^, ^11^ A total of 8442 and 7016 patients were randomized in the PARADIGM‐HF^9^ and ATMOSPHERE^8^ trials, respectively. The patients enrolled were in New York Heart Association (NYHA) functional class II–IV and had a left ventricular ejection fraction (EF) ≤35% (changed from ≤40% initially in PARADIGM‐HF by amendment) and elevated natriuretic peptide levels. In PARADIGM‐HF, patients were randomly assigned 1:1 to enalapril or sacubitril/valsartan. In ATMOSPHERE, patients were assigned 1:1:1 to enalapril, aliskiren or enalapril plus aliskiren. Data from PARADIGM‐HF and ATMOSPHERE were pooled for these analyses.

A total of 3023 and 4796 patients with HFpEF were enrolled in the CHARM‐Preserved^11^ and PARAGON‐HF^10^ trials, respectively. In CHARM‐Preserved, patients were in NYHA functional class II–IV and had an EF ≥40%. They were randomized 1:1 to candesartan or placebo. For this analysis, we excluded 450 patients from CHARM‐Preserved who had a left ventricular EF <45% to ensure a consistent EF threshold with PARAGON‐HF. In PARAGON‐HF, patients were in NYHA class II–IV and had an EF ≥45%, structural heart disease, and elevated natriuretic peptide levels. Patients were randomized 1:1 to treatment with either sacubitril/valsartan or valsartan. Data from PARAGON‐HF and CHARM‐Preserved were then pooled for these analyses.

For each of the trials, exclusion criteria with malignancy explicitly mentioned or implicitly relevant in relation to a life‐expectancy threshold were as follows – PARADIGM‐HF: life expectancy <5 years; ATMOSPHERE: life expectancy <5 years; PARAGON‐HF: life expectancy <3 years or any history of cancer within the previous 5 years; CHARM‐Preserved: life expectancy <2 years.

Case report forms for each of these trials recorded the presence or absence of a previous cancer diagnosis. Data on the time from diagnosis, site, stage, or histology of cancer were not captured. Patients with a prior history of cancer were compared with patients with no history of cancer. To determine the incidence of cancer during follow‐up, we examined new cancer diagnoses recorded as adverse events and serious adverse events in those without a history of cancer at baseline. Data were extracted and grouped based on the primary cancer site. Only patients with a clearly documented diagnosis of malignancy were included to avoid the inclusion of non‐malignant tumours or non‐melanomatous skin cancers.

### Clinical outcomes

The primary outcome in CHARM‐Preserved, PARADIGM‐HF and ATMOSPHERE was a composite of CV death or HF hospitalization in a time‐to‐first event analysis, while in PARAGON‐HF it was a composite of CV death and total (first and repeat) HF hospitalizations. In the current analysis, we examined the composite of CV death or HF hospitalization in each trial as well as its components, CV death and HF hospitalization, individually. We also examined death from any cause and non‐CV death. HF hospitalization and causes of death were adjudicated by a Clinical Events Committee according to standardized, pre‐specified criteria in each trial.^8^, ^9^, ^10^, ^11^

### Statistical analyses

Baseline characteristics are presented as means with standard deviations or medians with interquartile ranges for continuous variables, and frequencies with percentages for categorical variables. Patients were analysed according to history of cancer (baseline characteristics were compared using *t*‐tests, Wilcoxon rank‐sum tests, and chi‐squared tests where appropriate). The rates of all outcomes are presented per 100 patient‐years. Cox regression for time‐to‐first event was used to analyse each outcome and reported as the number of events and hazard ratios (HRs), with robust standard error accounting for clustering within trials, and the resulting 95% confidence intervals (CIs). Competing risk regression using the Fine–Grey method was used to analyse outcomes (to account for the risk of multiple potential competing events) and these outcomes are reported as sub‐distribution HRs (SHRs) with 95% CIs. The composite primary outcome and CV death were examined in the presence of the competing risk of non‐CV death, and first HF hospitalization was examined in the presence of the competing risk of all‐cause death. Non‐CV death was examined in the presence of the competing risk of CV death. HRs and SHRs are adjusted for the following variables, which were chosen based on clinical judgement: trial, randomized treatment, age, sex, region, heart rate, systolic blood pressure, body mass index, NYHA functional class, left ventricular EF, previous HF hospitalization, previous myocardial infarction, previous diabetes, smoking history, estimated glomerular filtration rate (eGFR), and N‐terminal pro‐B‐type natriuretic peptide (NT‐proBNP) (with missing indicator method used to handle missing eGFR and NT‐proBNP values).^12^ Poisson regression was used to analyse age‐ and sex‐adjusted incident cancer diagnoses, which are expressed per 100 patient‐years.

All analyses were performed using Stata version 16.0 (Stata Corp., College Station, TX, USA). Two‐sided *p*‐values <0.05 were considered significant.

## Results

### Baseline characteristics

Baseline characteristics of patients with and without a history of cancer are shown in *Table* *1*. Overall, there were 15 415 patients in the HFrEF trials (mean age 63.5 years) and 7363 patients in the HFpEF trials (mean age 70.6 years). There were 658 patients in the HFrEF trials with a history of cancer and 624 patients with a history of cancer in the HFpEF trials (4.3% and 8.5%, respectively). Overall, patients with HF and a history of cancer were on average 7.0 years older than those without. Patients with HFrEF and a history of cancer were 7.2 years older than those without a cancer history, while patients with HFpEF and a history of cancer were 4.3 years older than those without a cancer history (*Table* *1* and online supplementary *Table* *S1*).

**Table 1:** Baseline characteristics according to history of cancer in patients with heart failure with reduced ejection fraction (PARADIGM‐HF and ATMOSPHERE trials) and heart failure with preserved ejection fraction (PARAGON‐HF and CHARM‐Preserved trials)

Characteristic	Heart failure with reduced ejection fraction			Heart failure with preserved ejection fraction		
	No cancer (*n* = 14 757)	Cancer (*n* = 658)	*p*‐value	No cancer (*n* = 6739)	Cancer (*n* = 624)	*p*‐value
Age, years, mean ± SD	63.2 ± 11.6	70.4 ± 9.2	<0.001	70.3 ± 9.9	74.6 ± 8.2	<0.001
Age group, *n* (%)			<0.001			<0.001
<50 years	1769 (12.0)	17 (2.6)		172 (2.6)	4 (0.6)	
50–59 years	3397 (23.0)	58 (8.8)	824 (12.2)	29 (4.6)	
60–69 years	4856 (32.9)	198 (30.1)	1870 (27.7)	115 (18.4)	
≥70 years	4735 (32.1)	385 (58.5)	3873 (57.5)	476 (76.3)	
Male sex, *n* (%)	11 553 (78.3)	505 (76.7)	0.35	3500 (51.9)	302 (48.4)	0.091
Race, *n* (%)			<0.001			<0.001
Caucasian	9558 (64.8)	578 (87.8)		5682 (84.3)	572 (91.7)	
Black	526 (3.6)	11 (1.7)	186 (2.8)	24 (3.8)	
Asian	3232 (21.9)	41 (6.2)	648 (9.6)	23 (3.7)	
Other	1441 (9.8)	28 (4.3)	223 (3.3)	5 (0.8)	
HF aetiology, *n* (%)			0.007			
Ischaemic	8550 (57.9)	416 (63.2)				
Non‐ischaemic	6207 (42.1)	242 (36.8)				
SBP, mmHg, mean ± SD	122.4 ± 16.7	123.8 ± 17.4	0.026	132.9 ± 16.9	130.2 ± 16.3	<0.001
Weight category, *n* (%)			0.036			0.17
Underweight	296 (2.0)	11 (1.7)		28 (0.4)	6 (1.0)	
Normal	4402 (29.9)	163 (24.8)		1207 (17.9)	108 (17.4)	
Overweight	5601 (38.0)	269 (40.9)		2463 (36.6)	215 (34.6)	
Obese	4430 (30.1)	214 (32.6)		3034 (45.1)	293 (47.1)	
Comorbidities, *n* (%)						
Atrial fibrillation (history)	5193 (35.2)	288 (43.8)	<0.001	2957 (43.9)	331 (53.0)	<0.001
Hypertension	9805 (66.4)	467 (71.0)	0.016	5721 (84.9)	550 (88.1)	0.029
Myocardial infarction	6167 (41.8)	314 (47.7)	0.003	1991 (29.5)	146 (23.4)	0.001
Prior PCI or CABG	4406 (29.9)	279 (42.4)	<0.001	1953 (29.0)	194 (31.1)	0.27
Stroke	1151 (7.8)	66 (10.0)	0.038	670 (9.9)	60 (9.7)	0.83
Diabetes	4617 (31.3)	234 (35.6)	0.021	2554 (37.9)	236 (37.8)	0.97
COPD or asthma^a^	2104 (14.3)	133 (20.2)	<0.001	814 (18.7)	105 (24.2)	0.005
Current or ex‐smoker	6910 (46.8)	340 (51.7)	0.015	1970 (29.4)	212 (34.0)	0.015
HF characteristics, signs and symptoms						
Prior HF hospitalization, *n* (%)	9082 (61.5)	380 (57.8)	0.051	3734 (55.4)	319 (51.1)	0.039
NYHA functional class, *n* (%)			0.46			0.003
I	539 (3.7)	23 (3.5)		124 (1.8)	12 (1.9)	
II	10 282 (69.7)	477 (72.6)	4881 (72.5)	409 (65.5)	
III	3808 (25.8)	153 (23.3)	1676 (24.9)	197 (31.6)	
IV	116 (0.8)	4 (0.6)	56 (0.8)	6 (1.0)	
KCCQ clinical summary score, median (Q1–Q3)^a^ ^,^ ^b^	79.2 (62.5–91.7)	78.1 (62.5–90.6)	0.2	75.0 (60.4–87.5)	72.9 (58.1–84.4)	0.02
Dyspnoea on effort, *n* (%)	18 930 (88.2)	1153 (90.1)	0.098	6331 (94.0)	585 (93.9)	0.9
Dyspnoea at rest, *n* (%)	935 (4.4)	60 (4.7)	0.49	341 (5.1)	42 (6.7)	0.071
Orthopnoea, *n* (%)	2162 (10.1)	207 (16.2)	0.8	1234 (18.3)	164 (26.3)	<0.001
Paroxysmal nocturnal dyspnoea, *n* (%)	1168 (5.4)	84 (6.6)	0.38	449 (6.7)	47 (7.5)	0.41
Oedema, *n* (%)	5365 (25.0)	423 (33.0)	0.081	2327 (34.6)	269 (43.1)	<0.001
Third heart sound, *n* (%)	1540 (7.2)	73 (5.7)	0.25	202 (3.0)	22 (3.5)	0.46
JVD, *n* (%)	2148 (10.0)	172 (13.5)	0.49	729 (10.9)	114 (18.4)	<0.001
Investigations and management						
Ejection fraction, %, mean ± SD	37.7 ± 14.6	43.2 ± 15.7	0.026	57.0 ± 8.2	57.7 ± 8.0	0.043
NT‐proBNP, pg/ml, median (Q1–Q3)^a^						
History of AF	1698 (968–3091)	2045 (1213–3605)	0.001	1282 (735–1929)	1336 (710–2181)	0.3
No history of AF	1244 (693–2519)	1313 (754–2854)	0.14	578 (371–1032)	583 (360–965)	0.82
eGFR, ml/min/1.73 m^2^, mean ± SD	69 ± 22	62 ± 19	<0.001	64 ± 21	61 ± 20	<0.001
Loop diuretic, *n* (%)	11 808 (80.0)	528 (80.2)	0.89	5887 (87.4)	565 (90.5)	0.021
ACE inhibitor/ARB, *n* (%)	14 729 (99.8)	657 (99.8)	0.83	5155 (76.5)	492 (78.8)	0.18
Beta‐blocker, *n* (%)	13 649 (92.5)	594 (90.3)	0.036	4744 (70.4)	450 (72.1)	0.37
MRA, *n* (%)	7038 (47.7)	235 (35.7)	<0.001	1406 (20.9)	115 (18.4)	0.15
Digitalis, *n* (%)	4630 (31.4)	151 (22.9)	<0.001	1036 (15.4)	98 (15.7)	0.83
Pacemaker, *n* (%)	1662 (11.3)	138 (21.0)	<0.001	566 (8.4)	81 (13.0)	<0.001
ICD (including CRT‐D), *n* (%)	2139 (14.5)	152 (23.1)	<0.001	32 (0.5)	3 (0.5)	0.98

ACE, angiotensin‐converting enzyme; AF, atrial fibrillation; ARB, angiotensin receptor blocker; BMI, body mass index; CABG, coronary artery bypass grafting; COPD, chronic obstructive pulmonary disease; CRT‐D, cardiac resynchronization therapy‐defibrillator; DBP, diastolic blood pressure; eGFR, estimated glomerular filtration rate; HF, heart failure; ICD, implantable cardioverter‐defibrillator; JVD, jugular venous distension; KCCQ, Kansas City Cardiomyopathy Questionnaire; MRA, mineralocorticoid receptor antagonist; NT‐proBNP, N‐terminal pro‐B‐type natriuretic peptide; NYHA, New York Heart Association; PCI, percutaneous coronary intervention; SBP, systolic blood pressure; SD, standard deviation.

^a^ Only PARAGON‐HF for HFpEF patients (433 with cancer history, 4357 without).

^b^ Missing from HFrEF patients: 1862.

#### Comorbidities

Compared to HFrEF patients without a history of cancer, those with a history of cancer were more likely to be overweight and to have another comorbidity including atrial fibrillation (43.8% vs. 35.2%), hypertension (71.0% vs. 66.4%), previous myocardial infarction (47.7% vs. 41.8%), percutaneous coronary intervention or coronary artery bypass grafting (42.4% vs. 29.9%), stroke (10.0% vs. 7.8%) and diabetes (35.6% vs. 31.3%) (*Table* *1*). Similarly, HFpEF patients with a history of cancer were more likely to have atrial fibrillation (53.0% vs. 43.9%) and hypertension (88.1% vs. 84.9%) than those without a cancer history (*Table* *1*).

#### Heart failure characteristics and investigations

There were no differences in HF symptom burden or markers of congestion between HFrEF patients with and without a history of cancer. HFrEF patients with a history of cancer had a higher EF than those with no cancer history (43.2% vs. 37.7%).

By contrast, when compared to HFpEF patients without a history of cancer, those with a history of cancer were less likely to have a previous HF hospitalization (51.1% vs. 55.4%) but were more symptomatic with a higher prevalence of orthopnoea (26.3% vs. 18.3%) and more markers of congestion such as peripheral oedema (43.1% vs. 34.6%) and jugular venous distension (18.4% vs. 10.9%).

#### Background treatment

Patients with HFrEF and a history of cancer were less likely to receive treatment with beta‐blockers, mineralocorticoid receptor antagonists (MRA) and digoxin compared to those without. However, those with a cancer history were more likely to have a pacemaker (21.0% vs. 11.3%) or an implantable cardioverter‐defibrillator (23.1% vs. 14.5%) than those without (*Table* *1*). Patients with HFpEF with a history of cancer were more likely to be receiving diuretics (90.5% vs. 87.4%) and to have a pacemaker (13.0% vs. 8.4%) than those with no cancer history (*Table* *1*).

### Clinical outcomes

**Figure 1:**
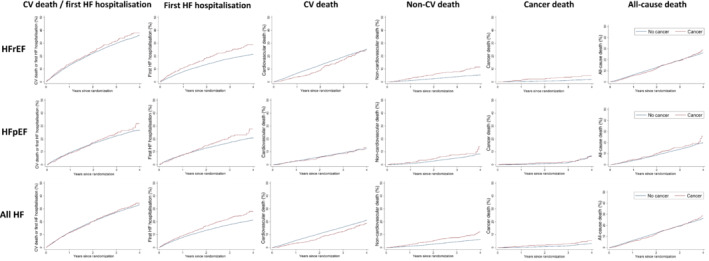
Outcomes in patients with and without a history of cancer in patients with heart failure with reduced ejection fraction (HFrEF) in the PARADIGM‐HF and ATMOSPHERE trials, heart failure with preserved ejection fraction (HFpEF) in the PARAGON‐HF and CHARM‐Preserved trials and all heart failure (HF) trials combined. The panels show the primary composite outcome, HF hospitalization, cardiovascular (CV) death, non‐CV death and all‐cause death.

The risk of the primary composite outcome of CV death and first HF hospitalization did not differ between patients with or without a history of cancer in either those with HFrEF (adjusted HR 1.03; 95% CI 0.89–1.20, *p* = 0.682) or HFpEF (adjusted HR 0.83; 95% CI 0.67–1.03, *p* = 0.086, respectively) (*Tables* *2*, *3*, *Figure* *1*). HFrEF patients with a history of cancer had a higher risk of a first hospitalization for HF with an adjusted HR of 1.28 (95% CI 1.07–1.52, *p* = 0.007) than those without (*Table* *2*). There was no difference in first HF hospitalization between HFpEF patients with and without a cancer history (adjusted HR 0.95; 95% CI 0.76–1.20, *p* = 0.681) (*Table* *3*).

**Table 2:** Outcomes according to a cancer history in patients with heart failure with reduced ejection fraction in the PARADIGM‐HF and ATMOSPHERE trials

Outcome	Total events		Events per 100 person‐years (95% CI)		Cancer vs. no cancer	
	No cancer	Cancer	No cancer	Cancer	Unadjusted^a^	Adjusted^b^
Primary composite outcome^c^	4206	194	11.89 (11.53–12.16)	12.97 (11.26–14.92)	1.12 (0.97–1.29) 0.137	1.03 (0.89–1.20) 0.682
First HF hospitalization	2377	142	6.72 (6.46–7.00)	9.49 (8.05–11.19)	1.38 (1.16–1.64) < 0.001	1.28 (1.07–1.52) 0.007
CV death	2764	108	7.19 (6.93–7.47)	6.35 (5.25–7.66)	0.94 (0.77–1.14) 0.532	0.85 (0.70–1.04) 0.106
Non‐CV death	518	51	1.35 (1.24–1.47)	3.00 (2.28–3.94)	1.92 (1.44–2.57) < 0.001	1.57 (1.16–2.12) 0.003
Cancer death	157	23	0.41 (0.35–0.48)	1.35 (0.90–2.03)	3.01 (1.93–4.69) < 0.001	2.03 (1.45–3.64) < 0.001
All‐cause death	3282	159	8.54 (8.25–8.84)	9.34 (8.00–10.91)	1.12 (0.96–1.32) 0.153	1.00 (0.85–1.18) 0.983

Hazard ratios are reported with 95% CIs within parentheses followed by *p*‐value.

CI, confidence interval; CV, cardiovascular; HF, heart failure.

^a^ Unadjusted analysis was adjusted for randomized treatment and region.

^b^ Adjusted for: age, sex, region, heart rate, systolic blood pressure, body mass index, N‐terminal pro B‐type natriuretic peptide, New York Heart Association functional class, ejection fraction, estimated glomerular filtration rate, previous hospitalization for HF, prior myocardial infarction or diabetes, and smoking history. Missing indicator method used to handle missing estimated glomerular filtration rate and N‐terminal pro‐B‐type natriuretic peptide.

^c^ CV death and HF hospitalization.

**Table 3:** Outcomes according to a cancer history in patients with heart failure with preserved ejection fraction in the CHARM‐Preserved and PARAGON‐HF trials

Outcome	Total events		Events per 100 person‐years (95% CI)		Cancer vs. no cancer	
	No cancer	Cancer	No cancer	Cancer	Unadjusted^a^	Adjusted^b^
Primary composite outcome^c^	1515	152	8.46 (8.05–8.90)	9.30 (7.94–10.91)	1.01 (0.85–1.20) 0.902	0.83 (0.67–1.03) 0.086
1st HF hospitalisation	1137	129	6.35 (5.99–6.73)	7.90 (6.64–9.38)	1.10 (0.92–1.33) 0.296	0.95 (0.76–1.20) 0.681
CV Death	640	56	3.27 (3.03–3.53)	3.12 (2.40–4.05)	0.96 (0.73–1.26) 0.750	0.72 (0.50–1.05) 0.085
Non‐CV death	356	49	1.82 (1.64–2.02)	2.73 (2.06–3.61)	1.46 (1.08–1.97) 0.015	1.09 (0.75–1.59) 0.639
Cancer death	159	17	0.81 (0.70–0.95)	0.95 (0.59–1.52)	1.45 (0.88–2.42) 0.147	1.16 (0.59–2.27) 0.670
All‐cause death	996	105	5.09 (4.78–5.42)	5.85 (4.83–7.08)	1.14 (0.93–1.40) 0.208	0.87 (0.67–1.13) 0.306

Hazard ratios are reported with 95% CIs within parentheses followed by *p*‐value.

CI, confidence interval; CV, cardiovascular; HF, heart failure.

^a^ Unadjusted analysis was adjusted for randomized treatment and region.

^b^ Adjusted for: age, sex, region, heart rate, systolic blood pressure, body mass index, N‐terminal pro‐B‐type natriuretic peptide, New York Heart Association functional class, ejection fraction, estimated glomerular filtration rate, previous hospitalization for HF, prior myocardial infarction or diabetes, and smoking history. Missing indicator method used to handle missing estimated glomerular filtration rate and N‐terminal pro‐B‐type natriuretic peptide.

^c^ CV death and HF hospitalization.

There was no association between cancer history and CV death in HFrEF or HFpEF patients (adjusted HR 0.85; 95% CI 0.70–1.04, *p* = 0.106 and adjusted HR 0.72; 95% CI 0.50–1.05, *p* = 0.085, respectively) (*Tables* *2*, *3*). The risk of non‐CV death was higher in HFrEF patients with a history of cancer (adjusted HR 1.57; 95% CI 1.16–2.12, *p* = 0.003) (*Table* *2*), but there was no difference in risk of non‐CV death in HFpEF patients with a cancer history (adjusted HR 1.09; 95% CI 0.75–1.59, *p* = 0.639) (*Table* *3*). Consequently, there was no association between cancer history and all‐cause mortality in either HFrEF patients (adjusted HR 1.00; 95% CI 0.85–1.18, *p* = 0.983) or HFpEF patients (adjusted HR 0.87; 95% CI 0.67–1.13, *p* = 0.306). The risk of cancer death was higher in HFrEF patients with a history of cancer than those without (adjusted HR 2.03; 95% CI 1.45–3.64, *p* < 0.001) (*Table* *2*), but there was no difference in the risk of cancer death in HFpEF patients with a cancer history (adjusted HR 1.16; 95% CI 0.59–2.27, *p* = 0.670) (*Table* *3*). These findings were similar when considering all HF patients together (online supplementary *Table* *S2*). After accounting for the competing risks of non‐CV death for the primary outcome and CV death, all‐cause death for first HF hospitalization, and CV death for non‐CV death, the associations were similar for HFrEF and HFpEF patients (online supplementary *Table* *S3*).

### Incident cancer diagnosis

**Figure 2:**
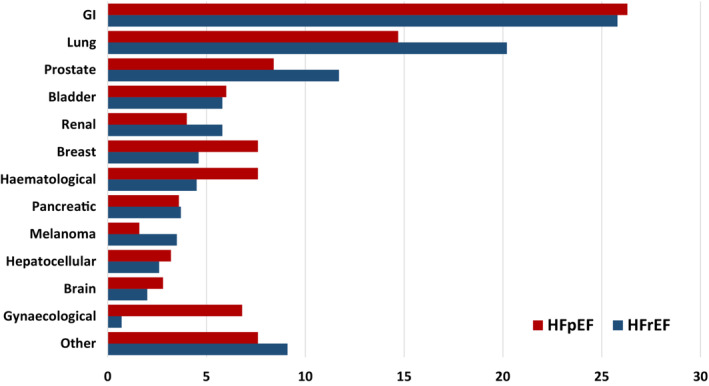
New cancer diagnoses by primary site in heart failure with reduced (HFrEF) and preserved ejection fraction (HFpEF) clinical trials (PARADIGM‐HF, ATMOSPHERE, PARAGON‐HF and CHARM‐Preserved). GI, gastrointestinal.

The numbers of new cancers reported during trial follow‐up are summarized in *Figure* *2*. Those with a history of cancer were excluded from this analysis to ensure only new cancers were analysed. There were 789 reports of cancer: 538 in the HFrEF trials (PARADIGM‐HF and ATMOSPHERE), and 251 in the HFpEF trials (PARAGON‐HF and CHARM‐Preserved). The incidence of new cancer in the HFrEF trials was 1.36 (95% CI 1.25–1.48) per 100 person‐years and 1.19 (95% CI 1.05–1.34) per 100 person‐years in the HFpEF trials. After adjusting for age and sex, the incidence of reported cancer in the HFrEF trials was 1.09 per 100 person‐years (95% CI 0.83–1.36) and 1.07 per 100 person‐years (95% CI 0.81–1.32) in the HFpEF trials. The overall incidence of new cancer in HF trials was 1.30 per 100 person‐years (95% CI 1.21–1.39). The most common cancers in both trials were gastrointestinal, lung and prostate, with smaller proportions of patients diagnosed with renal, pancreatic, hepatocellular, and haematological cancer. Gynaecological cancers accounted for a greater proportion of incident cancers in HFpEF than HFrEF patients (6.8% vs. 0.7%), but the frequency of other malignancies was similar between groups (*Figure* *2*).

## Discussion

In this analysis of patients with HF enrolled in clinical trials, we found that a history of cancer was associated with a higher risk of HF hospitalization and non‐CV death in HFrEF trial participants. This association persisted even after adjusting for differences in characteristics between those with and without cancer. However, there was no difference in all‐cause death because a higher risk of non‐CV death was offset by a trend to a lower risk of CV death. A history of cancer was not associated with any difference in clinical outcomes in HFpEF patients after adjusting for baseline characteristics. These findings may have implications for future HF trial design.

Compared to the general population, cancer survivors have a higher risk of CV disease, including HF.^13^, ^14^ As cancer survivorship grows, understanding the efficacy and safety of HF therapies in this population is increasingly important. Guidelines or position statements addressing the management of CV problems in cancer survivors are often based on limited evidence.^15^, ^16^ The number of patients with cancer in the trials underpinning guideline recommendations for angiotensin‐converting enzyme inhibitors, beta‐blockers and MRA in HFrEF has not been reported.^17^, ^18^, ^19^, ^20^, ^21^, ^22^ However, at the time those trials were conducted, it is likely that malignancy was widely considered to be a life‐limiting condition, thus excluding those patients (online supplementary *Table* *S4*). In the context of modern cancer therapy and dramatic improvements in survival for a wide range of malignancies,^23^ recent trials have refined exclusion criteria making many patients with a history of cancer eligible for participation. While there is some variability in the specifics of cancer‐related inclusion and exclusion criteria in these trials, patients with a history of cancer remain under‐represented: up to 20% of ‘real‐world’ patients with HF also have a history of cancer^4^, ^5^ while 5.6% of patients recruited into the trials we examined here had a past history of cancer. Irrespective of formal exclusion criteria, it is conceivable that investigators are less likely to consider discussing HF trial participation with patients who have a history of cancer. Whether patients who have had cancer are more or less likely to consent to participation in HF trials is also not known. In addition to the under‐representation of cancer patients in these trials, cancer mortality is not routinely reported. Furthermore, there is a ubiquitous lack of information relating to the class of anti‐cancer therapies received by patients with previous cancer in the trials analysed here, as well as by those in other contemporary HF trials (online supplementary *Table* *S4*). Given the recent rapid advances in the development of anti‐cancer drugs which exert both anti‐neoplastic and potential cardiotoxic effects via a broad range of mechanisms, recording this information is essential to refining our understanding of the implications of cancer treatments upon HF outcomes.

The higher risk of first HF hospitalization in HFrEF trial participants with cancer may be due to differences in baseline demographics, but the association persisted after extensive adjustment, including for natriuretic peptides. However, HFrEF trial participants with a cancer history were less likely to receive HF therapies, including beta‐blockers, MRAs and digoxin than those with no cancer history. This may explain why these patients were at higher risk of HF hospitalization. Additionally, there were no differences in HF therapies in HFpEF trial participants with and without cancer and no differences in HF hospitalization. Although the risk of non‐CV death was only higher in HFrEF trial participants with cancer, the risk of non‐CV death in all HFpEF trial participants was so high that it likely obscures any enhanced risk from a history of cancer in these patients. Although we demonstrated a potentially unsurprising higher risk of cancer death in HFrEF trial participants, this finding was not replicated in HFpEF trial participants. However, this analysis was under‐powered.

How might these findings be used to inform future trial design and analysis? Given the finding that there were higher rates of HF hospitalizations in trial participants with HFrEF and a history of cancer, better representation of patients with cancer may help with event accrual. This is particularly relevant in HFpEF where recurrent HF hospitalizations are a feature of the disease and the inclusion of recurrent events into the primary outcome of recent trials of therapies in HFpEF such as PARAGON‐HF^10^ has become commonplace and is increasingly used in HFrEF trials.^24^, ^25^ However, this would require HF treatments to be as effective at reducing HF hospitalizations in patients with cancer as those without, which is an area where data remain scarce. These findings may be counterintuitive to the previous view that a higher risk of non‐CV death among patients with cancer would lower the rates of modifiable non‐fatal and fatal events in trials. However, in our HFpEF cohort there was no association between cancer history and HF hospitalizations or CV death and the association with higher non‐CV death disappeared after adjustment. It would therefore appear that the inclusion of patients with cancer, as allowed by the protocols of these trials, and implemented by investigators, would have little effect on the planned event rates of a composite outcome in patients with HF. In HFrEF, their inclusion may in fact lead to a higher rate of events that are modifiable by a new therapy for HF which we would expect to reduce HF hospitalizations. Finally, their inclusion would make the results of future trials more generalizable to the population with HF in the community. In this study, there was no difference in all‐cause mortality between patients with and without a cancer history.

Survival in patients with HF continues to improve and current guideline‐recommended therapies lead to substantial improvements in lifetime survival, at least in HFrEF. Therefore, this growing population surviving with HF is at risk of developing cancer during their lifetime.^26^ Furthermore, there has been increasing interest in the potential for HF to be a pro‐oncogenic state.^27^ Given the potential pathophysiological links between HFpEF and cancer such as obesity, diabetes and inflammation,^28^ it may be expected that patients with HFpEF would have a higher incidence of cancer than those with HFrEF. However, we observed a similar incidence of new cancer diagnoses in trial participants with HFrEF and HFpEF when adjusted for age and sex (1.09 vs. 1.07 per 100 person‐years, respectively). Previously non‐randomized, observational studies suggested that angiotensin receptor blockers were associated with a higher risk of cancer.^29^ We only observed 34 cases of incident cancer in the one placebo group that was available in CHARM‐Preserved. This trial had been part of a much larger meta‐analysis of the risk of cancer with angiotensin receptor blockers which found no evidence of an increase in risk with this class of drugs,^30^ a finding which was supported by regulatory agency review.^30^, ^31^ The overall cancer incidence of 1.30 per 100 patient‐years observed here should be interpreted with some caveats, including the relatively brief trial follow‐up as well as non‐standardized screening for cancer. Additionally, participants with a history of cancer prior to trial enrolment were not included in this part of the analysis as it was not possible to determine if incident cancer was recurrent disease or a *de novo* diagnosis in these people. However, the cancer incidence we observed in the trial participants is similar to that seen in patients with HF in a Danish non‐trial participant population.^32^, ^33^ In that Danish study examining the incidence of cancer in 9307 HF patients (27% female, mean age 68 years, 89.3% HFrEF), the incidence of cancer was 1.89 per 100 patient‐years, while its incidence was 0.63 per 100 patient‐years in a matched population without HF.^32^ In an Italian analysis of administrative health data, the incidence of cancer in patients with HF was higher than seen here, at 2.1 per 100 patient‐years and this risk of cancer was 1.8 times more common than it was in the matched, non‐HF population. Cancer mortality was approximately four‐fold higher in HF patients than in control subjects.^34^

The main limitation of this analysis is that the history of cancer in patients enrolled in both the HFpEF and HFrEF trials did not specify the time from cancer diagnosis, the site of cancer, the staging, or the treatment patients received for it. The ascertainment of cancers during follow‐up relied on adverse event reports and was not a prospectively adjudicated outcome. Additionally, the patients enrolled in these trials had to meet other specific inclusion and exclusion criteria, and thus may not represent the wider HFrEF and HFpEF population and patients with cancer. Indeed, our analysis results are specifically relevant to patients with HF recruited to clinical trials and should not be considered to be a reflection of epidemiology in the wider, non‐trial participant population. Additionally, the lack of a control group of patients without HF mean these results cannot be compared with the general population. However, by virtue of being enrolled within clinical trials, these patients were well‐characterized at baseline and had rigorous, systematic follow‐up with adjudicated clinical outcomes.

In conclusion, we found that HFrEF patients enrolled in the ATMOSPHERE and PARADIGM‐HF trials with a history of cancer had higher risks of HF hospitalization and non‐CV death. HFrEF patients enrolled in ATMOPSHERE and PARADIGM‐HF, and HFpEF patients enrolled in PARAGON‐HF and CHARM‐Preserved with a history of cancer were more likely to be older and have higher levels of comorbidity. However, they had similar risks of CV and all‐cause mortality to those with no cancer history. This may have implications for clinical trial design. There was no difference in incident cancer diagnoses between patients with HFrEF and HFpEF.

**Conflict of interest**: none declared.

## Supporting information


**Appendix S1.** Supporting Information.
