# Supplementary material for: Characteristics and outcomes of patients with a history of cancer recruited to heart failure trials
Source: Eur J Heart Fail. 2023 Mar 15;25(4):488–96. doi: 10.1002/ejhf.2818 (PMC10947056; doi:10.1002/ejhf.2818)
Supplement: Supplementary file 1 — Appendix S1. Supporting Information. [file EJHF-25-488-s001.docx]

**APPENDIX**

**TABLE LEGENDS**

**Supplementary table 1.** Baseline characteristics in patients with heart failure (HFrEF and HFpEF combined) with and without a history of cancer in PARADIGM-HF, ATMOSPHERE, PARAGON AND CHARM-preserved trials.

**Supplementary table 2.** Outcomes according to history of cancer versus no history of cancer in all heart failure patients in PARADIGM-HF, ATMOSPHERE, PARAGON AND CHARM-preserved trials.

**Supplementary table 3.** Risk of outcomes according to a history of cancer in patients with HFrEF (PARADIGM-HF AND ATMOSPHERE trials) and HFpEF (PARAGON and CHARM-preserved trials) in a competing risk analysis.

**Supplementary Table 4.** Recording of cancer history and associated exclusion criteria in key heart failure clinical trials.

| **Supplementary Table 1.** Baseline characteristics in patients with heart failure (HFrEF and HFpEF combined), with and without a history of cancer, in the PARADIGM-HF, ATMOSPHERE, PARAGON-HF and CHARM-preserved trials. | | | |  |
| --- | --- | --- | --- | --- |
|  | No cancer N=21,496 | Cancer N=1,282 | p-value |  |
|  |  |  |  |  |
| Age ± SD | 65.5 ± 11.6 | 72.5 ± 9.0 | <0.001 |  |
|  |  |  |  |  |
| Age group, no. (%) |  | | <0.001 |  |
|  |  |  |  |  |
| <50 years | 1,941 (9.0) | 21 (1.6) |  |  |
| 50-59 years | 4,221 (19.6) | 87 (6.8) |  |  |
| 60-69 years | 6,726 (31.3) | 313 (24.4) |  |  |
| ≥70 years | 8,608 (40.0) | 861 (67.2) |  |  |
| Sex, no. (%) |  | | <0.001 |  |
|  |  |  |  |  |
| Men | 15,053 (70.0) | 807 (62.9) |  |  |
| Race, no. (%) |  | | <0.001 |  |
|  |  |  |  |  |
| White | 15,240 (71.1) | 1,150 (89.8) |  |  |
| Black | 712 (3.3) | 35 (2.7) |  |  |
| Asian | 3,880 (18.1) | 64 (5.0) |  |  |
| Other | 1,611 (7.5) | 32 (2.5) |  |  |
| HF aetiology, no. (%) |  | | 0.007 |  |
|  |  |  |  |  |
| Ischaemic | 8,550 (57.9) | 416 (63.2) |  |  |
| Non-ischaemic | 6,207 (42.1) | 242 (36.8) |  |  |
| SBP, mm Hg; mean ± SD | 126 ± 17 | 127 ± 17 | 0.01 |  |
|  |  |  |  |  |
| Weight category, no. (%) |  |  | <0.001 |  |
|  |  |  |  |  |
| Underweight | 324 (1.5) | 17 (1.3) |  |  |
| Normal | 5,609 (26.1) | 271 (21.2) |  |  |
| Overweight | 8,064 (37.6) | 484 (37.8) |  |  |
| Obese | 7,464 (34.8) | 507 (39.6) |  |  |
| **Comorbidities**, no. (%) |  | | |  |
|  |  |  |  |  |
| Atrial fibrillation (history) | 8,150 (37.9) | 619 (48.3) | <0.001 |  |
|  |  |  |  |  |
| Hypertension | 15,526 (72.2) | 1,017 (79.3) | <0.001 |  |
|  |  |  |  |  |
| Myocardial infarction | 8,158 (38.0) | 460 (35.9) | 0.14 |  |
|  |  |  |  |  |
|  |  |  |  |  |
| Prior PCI or CABG | 6,359 (29.6) | 473 (36.9) | <0.001 |  |
|  |  |  |  |  |
| Stroke | 1,821 (8.5) | 126 (9.9) | 0.085 |  |
|  |  |  |  |  |
| Diabetes | 7,171 (33.4) | 470 (36.7) | 0.015 |  |
|  |  |  |  |  |
| COPD or Asthma* | 2,918 (15.3) | 238 (21.8) | <0.001 |  |
|  |  |  |  |  |
| Current or ex-smoker | 8,880 (41.4) | 552 (43.1) | 0.22 |  |
|  |  |  |  |  |
| **Heart failure characteristics, signs and symptoms** | | | |  |
|  |  |  |  |  |
| Prior HF hospitalisation, no. (%) | 12,816 (59.6) | 699 (54.5) | <0.001 |  |
|  |  |  |  |  |
| NYHA functional class, no. (%) |  | | 0.5 |  |
|  |  |  |  |  |
| I | 663 (3.1) | 35 (2.7) |  |  |
| II | 15,163 (70.6) | 886 (69.2) |  |  |
| III | 5,484 (25.5) | 350 (27.3) |  |  |
| IV | 172 (0.8) | 10 (0.8) |  |  |
| KCCQ clinical summary score, median (Q1-Q3) * ¥ | 78.1  (62.0-90.6) | 76.0  (59.9-88.8) | 0.001 |  |
|  |  |  |  |  |
| Dyspnoea on effort | 18,930 (88.2) | 1,153 (90.1) | 0.039 |  |
|  |  |  |  |  |
| Dyspnoea at rest | 935 (4.4) | 60 (4.7) | 0.57 |  |
|  |  |  |  |  |
| Orthopnoea | 2,162 (10.1) | 207 (16.2) | <0.001 |  |
|  |  |  |  |  |
| Paroxysmal nocturnal dyspnoea | 1,168 (5.4) | 84 (6.6) | 0.087 |  |
|  |  |  |  |  |
| Oedema | 5,365 (25.0) | 423 (33.0) | <0.001 |  |
|  |  |  |  |  |
| Third heart sound | 1,540 (7.2) | 73 (5.7) | 0.048 |  |
|  |  |  |  |  |
| JVD | 2,148 (10.0) | 172 (13.5) | <0.001 |  |
|  |  |  |  |  |
| **Investigations and management**, no. (%) | | | |  |
|  |  |  |  |  |
| Ejection fraction (%), mean ± SD | 37.7 ± 14.6 | 43.2 ± 15.7 | <0.001 |  |
|  |  |  |  |  |
| NT-proBNP, pg/ml; median (Q1-Q3) * |  | |  |  |
|  |  |  |  |  |
| History of AF | 1528  (909 – 2648) | 1596  (929 – 2879) | 0.14 |  |
|  |  |  |  |  |
| No history of AF | 1078  (602 – 2212) | 998  (559 – 2119) | 0.24 |  |
|  |  |  |  |  |
| eGFR, mL/min/1.73m^2^, mean ± SD | 69 ± 22 | 62 ± 19 | <0.001 |  |
|  |  |  |  |  |
| Loop diuretic | 17,695 (82.3) | 1,093 (85.3) | 0.007 |  |
|  |  |  |  |  |
| ACE inhibitor / ARB | 19,884 (92.5) | 1,149 (89.6) | <0.001 |  |
|  |  |  |  |  |
| Beta-blocker | 18,393 (85.6) | 1,044 (81.4) | <0.001 |  |
|  |  |  |  |  |
| MRA | 8,444 (39.3) | 350 (27.3) | <0.001 |  |
|  |  |  |  |  |
| Digitalis | 5,666 (26.4) | 249 (19.4) | <0.001 |  |
|  |  |  |  |  |
| Pacemaker | 2,228 (10.4) | 219 (17.1) | <0.001 |  |
|  |  |  |  |  |
| ICD (including CRT-D) | 2,171 (10.1) | 155 (12.1) | 0.022 |  |
|  |  |  |  |  |

ACE – angiotensin-converting enzyme, ARB – angiotensin receptor blocker, BMI – body mass index, CABG – coronary artery bypass grafting, COPD – chronic obstructive pulmonary disease, CRT-D – cardiac resynchronisation therapy-defibrillator, DBP – diastolic blood pressure, eGFR – estimated glomerular filtration rate, HF – heart failure, ICD – implantable cardioverter defibrillator, JVD – jugular venous distension, KCCQ – Kansas City Cardiomyopathy Questionnaire, MRA – mineralocorticoid receptor antagonist, NT-proBNP – N-terminal pro B-type natriuretic peptide, NYHA – New York Heart Association, PCI – percutaneous coronary intervention, SBP – systolic blood pressure.

*Only PARAGON-HF for HFpEF patients (433 with cancer history, 4357 without).

¥ Missing from HFrEF patients: 1862.

| **Supplementary Table 2**. Outcomes according to a history of cancer, versus no history of cancer, in all heart failure patients in the PARADIGM-HF, ATMOSPHERE, PARAGON-HF and CHARM-Preserved trials. | | | | | | |  |
| --- | --- | --- | --- | --- | --- | --- | --- |
|  | Total events | | Events per 100 person-years (95% CI) | | Cancer vs No cancer | |  |
|  | No cancer | Cancer | No cancer | Cancer | Unadjusted† | Adjusted‡ |  |
| Primary composite outcome* | 5721 | 346 | 10.74 (10.47 - 11.02) | 11.05 (9.95 - 12.28) | 1.12 (0.97 - 1.29) 0.137 | 1.03 (0.89 - 1.20) 0.682 |  |
|  |  |  |  |  |  |  |  |
| 1st HF hospitalisation | 3514 | 271 | 6.60 (6.38 - 6.82) | 8.66 (7.69 - 9.75) | **1.28 (1.13 - 1.45) <0.001** | **1.16 (1.01 - 1.33) 0.037** |  |
|  |  |  |  |  |  |  |  |
| CV Death | 3404 | 164 | 5.87 (5.68 - 6.07) | 4.69 (4.02 - 5.47) | 0.94 (0.80 - 1.10) 0.452 | **0.81 (0.68 - 1.02) 0.019** |  |
|  |  |  |  |  |  |  |  |
| Non-CV death | 874 | 100 | 1.51 (1.41 - 1.61) | 2.86 (2.35 - 3.48) | **1.65 (1.34 - 2.04) <0.001** | **1.33 (1.05 - 1.68) 0.017** |  |
|  |  |  |  |  |  |  |  |
| Cancer death | 316 | 40 | 0.54 (0.49 – 0.61) | 1.14 (0.84 – 1.56) | **2.00 (1.43 – 2.79) <0.001** | **1.76 (1.21 – 2.58) 0.003** |  |
| All-cause death | 4278 | 264 | 7.38 (7.16 - 7.60) | 7.55 (6.69 - 8.52) | 1.12 (0.99 - 1.27) 0.072 | 0.95 (0.83 - 1.10) 0.498 |  |
|  |  |  |  |  |  |  |  |

Hazard ratios are reported with 95% CIs within parentheses followed by p-value.

* CV death and HF hospitalisation

† unadjusted analysis was adjusted for randomised treatment and region.

‡ adjusted variables include age, sex, region, heart rate, systolic blood pressure, body mass index, NT-proBNP, NYHA functional class, ejection fraction, eGFR, previous hospitalisation for HF, prior MI or diabetes, and smoking history. Missing indicator method used to handle missing eGFR and NT-proBNP.

| **Supplementary Table 3**. Risk of outcomes according to a history of cancer in patients with HFrEF (PARADIGM-HF AND ATMOSPHERE trials) and HFpEF (PARAGON-HF and CHARM-preserved trials) in a competing risk analysis. | | | | | | |  |
| --- | --- | --- | --- | --- | --- | --- | --- |
|  | All HF | | HFrEF | | HFpEF | |  |
|  |  |  |  |  |  |  |  |
|  | Cancer vs No cancer | | Cancer vs No cancer | | Cancer vs No cancer | |  |
|  | Unadjusted† | Adjusted‡ | Unadjusted† | Adjusted‡ | Unadjusted† | Adjusted‡ |  |
| Primary composite outcome* | 1.07 (0.96 - 1.19) 0.246 | 0.96 (0.85 - 1.09) 0.543 | 1.08 (0.93 - 1.25) 0.322 | 1.02 (0.88 - 1.20) 0.770 | 1.00 (0.85 - 1.18) 0.969 | 0.82 (0.66 - 1.01) 0.058 |  |
|  |  |  |  |  |  |  |  |
| 1st HF hospitalisation | **1.30 (1.15 - 1.47) <0.001** | **1.20 (1.04 - 1.38) 0.012** | **1.29 (1.08 - 1.53) 0.004** | **1.26 (1.05 - 1.51) 0.013** | 1.10 (0.92 - 1.32) 0.313 | 0.95 (0.76 - 1.19) 0.680 |  |
|  |  |  |  |  |  |  |  |
| CV Death | 0.89 (0.77 - 1.04) 0.156 | 0.80 (0.67 – 0.95) 0.013 | 0.95 (0.78 - 1.15) 0.589 | 0.87 (0.72 - 1.07) 0.185 | 0.94 (0.72 - 1.24) 0.666 | 0.72 (0.50 – 1.04) 0.076 |  |
|  |  |  |  |  |  |  |  |
| Non-CV death | **1.69 (1.37 - 2.09) <0.001** | **1.39 (1.09 - 1.77) 0.008** | **1.97 (1.47 - 2.65) <0.001** | 1.14 (0.78 - 1.69) 0.498 | **1.47 (1.08 - 2.00) 0.015** | 1.14 (0.78 - 1.69) 0.498 |  |
|  |  |  |  |  |  |  |  |

Results are displayed as subdistribution hazard ratios (SHRs) with 95% CIs within parentheses followed by p-value.

All outcomes were tested for competing risks of all-cause and non-cardiovascular death. Non-CV death was tested for the competing risk of CV death. A missing indicator method was used to handle missing eGFR and NT-proBNP values.

* CV death and HF hospitalisation

† unadjusted analysis was adjusted for randomised treatment and region.

‡ adjusted variables include age, sex, region, heart rate, systolic blood pressure, body mass index, NT-proBNP, NYHA functional class, ejection fraction, eGFR, previous hospitalisation for HF, prior MI or diabetes, and smoking history

| **Supplementary Table 4.** Recording of cancer history and associated exclusion criteria in key heart failure clinical trials. | | | | | | |  |
| --- | --- | --- | --- | --- | --- | --- | --- |
| **Trial** | **Cancer history** | **Cancer site** | **Time from diagnosis** | **Cancer treatment** | **Exclusion criteria with malignancy explicitly mentioned or implied** |  |  |
|  |  |  |  |  |  |  | |
|  |  |  |  |  |  |  | |
| **Angiotensin receptor / neprilysin inhibitors** | | | | | |  | |
|  |  |  |  |  |  |  | |
| PARADIGM-HF | Yes | Yes | Yes | No | Life expectancy < 5 years |  | |
|  |  |  |  |  |  |  | |
| PARAGON-HF | Yes | No | Yes | No | Life expectancy < 3 years or any history of cancer within prior 5 years |  | |
|  |  |  |  |  |  |  | |
| **Angiotensin-converting enzyme inhibitors / angiotensin receptor antagonists** | | | | | |  | |
|  |  |  |  |  |  |  | |
| CHARM-Preserved | Yes | Yes | No | No | Life expectancy <2 years |  | |
|  |  |  |  |  |  |  | |
| SOLVD-T | No | No | No | No | History of cancer within prior 5 years |  | |
|  |  |  |  |  |  |  | |
| CONSENSUS | UNK | UNK | UNK | UNK | Not specified |  | |
|  |  |  |  |  |  |  | |
| **Beta blockers** | | | | | |  | |
|  |  |  |  |  |  |  | |
| CIBIS II | UNK | UNK | UNK | UNK | Life-threatening diseases (including malignancy) |  | |
|  |  |  |  |  |  |  | |
| MERIT-HF | UNK | UNK | UNK | UNK | Any other serious disease that might complicate management and follow up |  | |
|  |  |  |  |  |  |  | |
| COPERNICUS | UNK | UNK | UNK | UNK | Not specified |  | |
|  |  |  |  |  |  |  | |
| SENIORS | UNK | UNK | UNK | UNK | Any medical condition that may reduce survival during the study |  | |
|  |  |  |  |  |  |  | |
| **Mineralocorticoid receptor antagonists** | | | | | |  | |
|  |  |  |  |  |  |  | |
| RALES | UNK | UNK | UNK | UNK | Active cancer or any life-threatening disease |  | |
|  |  |  |  |  |  |  | |
| EMPHASIS-HF | Yes | Yes | Yes | No | Pre-existing cancer Life expectancy < 3 years |  | |
|  |  |  |  |  |  |  | |
| **Sodium-glucose transporter-2 inhibitors** | | | | | |  | |
|  |  |  |  |  |  |  | |
| DAPA-HF | UNK | UNK | UNK | UNK | Active malignancy requiring treatment at time of visit 1 Life expectancy < 2 years |  | |
|  |  |  |  |  |  |  | |
| EMPEROR-Reduced | UNK | UNK | UNK | UNK | Active or suspected malignancy or history of malignancy within 2 years  Life expectancy < 1 year |  | |
|  |  |  |  |  |  |  | |
| **Renin inhibitors** | | | | | |  | |
|  |  |  |  |  |  |  | |
| ATMOSPHERE | Yes | Yes | Yes | No | Life expectancy < 5 years |  | |
|  |  |  |  |  |  |  | |
| **Ivabradine** | | | | | |  | |
|  |  |  |  |  |  |  | |
| SHIFT | UNK | UNK | UNK | UNK | Any non-cardiac disease (e.g., cancer) judged likely to limit  3-year survival |  | |
|  |  |  |  |  |  |  | |
| **Isosorbide dinitrate / hydralazine** | | | | | |  | |
|  |  |  |  |  |  |  | |
| V-HEFT | UNK | UNK | UNK | UNK | Malignancies likely to limit  5-year survival |  | |
|  |  |  |  |  |  |  | |
| A-HEFT | UNK | UNK | UNK | UNK | Presence of an illness other than HF that was likely to result in death within the study period |  | |
|  |  |  |  |  |  |  | |
| **Digoxin** | | | | | |  | |
|  |  |  |  |  |  |  | |
| DIG | UNK | UNK | UNK | UNK | Life expectancy < 3 years (e.g., most cancers) |  | |
|  |  |  |  |  |  |  | |

A-HEFT – African-American heart failure trial; ATMOSPHERE – The aliskiren trial to minimise outcomes in patients in patient with heart failure; CHARM-Preserved – Effects of candesartan in patients with chronic heart failure and preserved left ventricular ejection fraction; CIBIS II – The cardiac insufficiency bisoprolol study II; CONSENSUS – Cooperative north Scandinavian enalapril survival study; COPERNICUS – Carvedilol prospective randomised cumulative survival; DAPA-HF – Dapagliflozin and prevention of adverse outcomes in heart failure; DIG – Digitalis investigation group; EMPEROR-Reduced – Empagliflozin outcome trial in patients with chronic heart failure with reduced ejection fraction; EMPHASIS-HF – Effect of eplerenone versus placebo on cardiovascular mortality and heart failure hospitalisation in subjects with NYHA class II chronic systolic heart failure; MERIT-HF – Metoprolol CR/XL randomised intervention trail in congestive heart failure; PARADIGM-HF – Prospective comparison of ARNI with ACEi to determine impact on global mortality and morbidity in heart failure; PARAGON-HF – Prospective comparison of ARNI with ARB global outcomes in heart failure with preserved ejection fraction; RALES – Randomised Aldactone evaluation study; SENIORS – Study of effects of nebivolol intervention on outcomes and rehospitalisation in seniors with heart failure; SHIFT – Ivabradine and outcomes in chronic heart failure; SOLVD-T – Studies of left ventricular dysfunction; V-HEFT – Vasodilator heart failure trial.
